# Phase-based masking for quantitative susceptibility mapping of the human brain at 9.4T

**DOI:** 10.1002/mrm.29368

**Published:** 2022-06-26

**Authors:** Gisela E. Hagberg, Korbinian Eckstein, Elisa Tuzzi, Jiazheng Zhou, Simon Robinson, Klaus Scheffler

**Affiliations:** 1 Department for Biomedical Magnetic Resonance, University Hospital Tübingen, Tübingen, Germany; 2 Department of Neurology, Medical University of Graz, Graz, Austria; 3 High Field MR Centre, Department of Biomedical Imaging and Image-Guided Therapy, Medical University of Vienna, Vienna, Austria; 4 Centre for Advanced Imaging, The University of Queensland, Brisbane, Australia; 5 High Field Magnetic Resonance Center, Max Planck Institute for Biological Cybernetics, Tübingen, Germany

**Keywords:** magnetic resonance imaging, magnetic susceptibility, MRI methods, QSM, tissue masking

## Abstract

**Purpose:**

To develop improved tissue masks for QSM.

**Methods:**

Masks including voxels at the brain surface were automatically generated from the magnitude alone (MM) or combined with test functions from the first (PG) or second (PB) derivative of the sign of the wrapped phase. Phase images at 3T and 9.4T were simulated at different TEs and used to generate a mask, *P_Itoh_*, with between-voxel phase differences less than π. MM, PG, and PB were compared with *P_Itoh_*. QSM were generated from 3D multi-echo gradient-echo data acquired at 9.4T (21 subjects aged: 20-56y), and from the QSM2016 challenge 3T data using different masks, unwrapping, background removal, and dipole inversion algorithms. QSM contrast was quantified using age-based iron concentrations.

**Results:**

Close to air cavities, phase wraps became denser with increasing field and echo time, yielding increased values of the test functions. Compared with *P_Itoh_*, PB had the highest Dice coefficient, while PG had the lowest and MM the highest percentage of voxels outside *P_Itoh_*. Artifacts observed in QSM at 9.4T with MM were mitigated by stronger background filters but yielded a reduced QSM contrast. With PB, QSM contrast was greater and artifacts diminished. Similar results were obtained with challenge data, evidencing larger effects of mask close to air cavities.

**Conclusion:**

Automatic, phase-based masking founded on the second derivative of the sign of the wrapped phase, including cortical voxels at the brain surface, was able to mitigate artifacts and restore QSM contrast across cortical and subcortical brain regions.

## 1 Introduction

QSM at high fields yield information about local tissue microstructure, myelin, iron content, calcifications, and venous oxygenation saturation^1–9^. QSM maps are derived from phase images acquired with a gradient echo sequence, after phase unwrapping^10–16^, background removal,^9,17–21^and dipole inversion^22–25^. The quality of different preprocessing steps can be verified using standardized challenge data sets^26,27^. QSM relies on adequate brain tissue masking, generally identified from the magnitude image, which is further eroded during background removal^28^. The performance of magnitude-based masking strategies is limited by inhomogeneous B_0_ and B_1_ fields that cause increased signal dropout at late TEs and image inhomogeneity, which lead to errors in the estimation at the brain boundary.

Accurate QSM quantification is problematic due to the presence of strong background fields that may cause multiple phase wraps, especially at high fields^29^. The wraps are frequent in the proximity of air cavities^30,31^ but can be unwrapped if the difference in the underlying phase between two neighboring voxels is less than π, known as ‘Itoh’s condition’^32^. In the absence of any MRI signal, the phase exhibits a uniform probability distribution between ±π^33^.

In the present work, we developed improved, fully automated masking strategies, aiming to maximize brain coverage by including voxels located close to the brain surface. Besides tissue masks from the magnitude image (MM), we also explored the combination with phase-based masks, obtained from the first (PG) and second (PB) derivative of the sign of the wrapped phase, followed by smoothing and thresholding. Field mapping at 3T provided the basis for simulations of phase behavior at different fields and echo times, that were used to generate tissue masks, and to verify Itoh’s condition. 3D gradient multi-echo images were measured in vivo at 9.4T (N = 21, age 20-56y). Performance of the masking strategies for generating QSM from these data and from the 3T data of the QSM2016 challenge^26^ were compared for different combinations of unwrapping, background removal, and dipole inversion algorithms, through assessments of the QSM contrast, quantified based on age- and region-specific estimates of cortical and subcortical iron concentrations.

## 2 Methods

### 2.1 MRI measurements and image reconstruction

Healthy volunteers (N = 22, 29 ± 11 y; eight females) were scanned at 9.4T (Siemens Healthineers, Erlangen, Germany) with a 16 channel transmit/31 channel receive array^34^ after providing written, informed consent, as approved by the Ethics Review Board of the Eberhard-Karl’s University, Tübingen.

Actual flip angle^35^ (FA = 60°; TR1/TR2 = 20/100 ms; TE = 7 ms, voxel size = 3 x 3 x 5 mm^3^) and MP2RAGE^36^ (TI1/TI2 = 900/3500 ms; FA = 4/6°; TR = 6 ms; volume TR = 8894 ms, 0.8 mm isotropic voxels) images were processed to yield tissue segmentations and regions of interest^37^ (ROI) as described previously^38^. Monopolar, multi-echo 3D gradient-echo (GRE) images (5 echoes, TE = 6-30 ms in steps of 6 ms; TR = 35 ms; FA = 11°; matrix: 512x464; FoV = 192x174mm; 96 partitions; 0.8 or 1 mm thick slices, GRAPPA = 2; partial Fourier acquisition (PFA) = 6/8; read-out bandwidth = 240 Hz/pixel) were acquired for QSM, with offline reconstruction of single echo images: k-space zero-filling to twice the acquired 3D matrix, Projection Onto Convex Sets reconstruction^39^, ASPIRE^13^ phase offset correction but without spatial smoothing of the phase offset maps, and adaptive channel combination based on the ASPIRE-corrected first echo image with a block size of one.^40^ Combination of three or five echoes was made with Fit_ppm_complex_TE^41–43^.

A field map (FM) was measured at 3T (PrismaFit, Siemens Healthineers, Erlangen, Germany) using the UTE sequence (TE = 0.1; 1.11; 2.22 ms; voxel size = [1.5 mm]^3^, Siemens WIP992D) to minimize phase wraps, albeit neglecting differences in eddy currents and shim with respect to 9.4T (N = 1). After reconstruction using scanner software and voxel-wise phase unwrapping in the TE domain, the FM was generated from the first two echoes, interpolated to match the 9.4T data and smoothed (Gaussian, full width half maximum, FWHM = 8 mm, Supporting Information Figure S1, which is available online) before simulating phase evolutions with echo time and field (Supporting Information Figure S2). The phase image with TE = 18 ms at 9.4T was used to identify a mask, *P_Itoh_* by thresholding $Test_{Ifoh}=\sqrt{G_{x}^{2}+G_{y}^{2}+G_{z}^{2}}$ (where *G_x_*, *G_y_*, and *G_z_* indicate the phase gradient along the x, y, and z direction, respectively) at a value of π.

### 2.2 Generation of tissue masks

Magnitude masking MM was obtained with FSL’s brain extraction tool^44^ (BET) with the -R option (and -B at 3T) and different values for the fractional intensity threshold FIT (−f 0.1–0.8 in steps of 0.1) followed by Gaussian smoothing (FWHM = 4 voxels) to include voxels close to the brain surface.

Phase-based masks from the first (PG) and second (PB) derivative of the sign, *S*, of the wrapped phase *wP* were generated in Matlab (Natick, MA, USA) in five steps:

step1: calculate *S* of *wP*;

step2: generate a) PG: norm of the gradient images of *S*; b) PB: absolute value of the Laplacian of *S*;

step3: smooth to obtain test functions in 3D, *Test^PG^* and *Test^PB^*;

step4: threshold and multiply with MM;

step5: close and open image (to fill holes within the mask, maintaining voxels at the brain surface).

Maximum values, *Test^PG^*_max_, *Test^PB^*_max_ were identified by simulations of phase noise in 3D for a matrix of 100x100x100 voxels. Steps 1–3 were applied, maximum values in the central 90x90x90 voxels extracted and explored using the ‘cftool’ in Matlab. The effects of smoothing with a sphere (radius, 1 ≤ *r* ≤ 8 voxels) and a Gaussian kernel (1 ≤ *FWHM* ≤ 8 voxels) on *Test_max_* were explored.

PG and PB masks were generated from the simulated phase with TE = 18 ms at 9.4T. The Dice coefficients between the ground truth *P_Itoh_* mask and PG or PB masks obtained by thresholding at different levels of *Test*^max^_*PG*_ and *Test*^max^_*PB*_ (25, 50, 75, and 100%) were calculated and the percentage of voxels included in PG or PB but outside *P_Itoh_* were determined. The same parameters were also evaluated for MM.

### 2.3 Generation of Quantitative Susceptibility Maps

Phase images were unwrapped using the Laplacian approach^10^ and ROMEO^14^.

Whole brain referencing consisted of subtraction of the median value of the unwrapped phase inside the tissue mask. The background field was removed using ‘sophisticated harmonic artifact reduction for phase data’ (SHARP^9^) algorithms, either RESHARP^17^ with kernel sizes between 0.8 and 4 mm and Tikhonov regularization parameters ranging from 10^−3^(Tk-3) to 10^−12^(Tk-12) or V-SHARP^20^ with a maximum value for the spherical mean value (SMV) between 4-20 mm. Alternatively, the Laplacian boundary value (LBV) method^21^ with a tolerance of 0.005 and other parameters set to default values in the MEDI toolbox^45^ was used.

Dipole inversion to obtain QSM maps was performed using iLSQR^20,46,47^ and MEDI^43^ with a lambda factor of 100.

### 2.4 Quantification of iron-dependent QSM contrast

QSM values were extracted from six cortical ROIs (with gray matter tissue probability>0.98) and three sub-cortical ROIs (eroded with a sphere of radius 2 voxels). The median QSM value was calculated for each ROI within the final MM or PB mask obtained after erosion during background removal.

For each subject and ROI, the expected age-dependent non-heme iron concentration was obtained from^48^:

(Eq.1) $$[Fe]=A\cdot \left(1-e^{-B\cdot age}\right)+C$$

using ROI-dependent coefficients A, B, and C (Supporting Information Table S1).

Linear regression analysis:

(Eq.2) $$QSM=k_{Fe}\cdot [Fe]+\chi_{\text{other}}$$

yielded *k_Fe_*, (unit [ppb/μg/g]) to quantify the iron-dependent QSM contrast, with *χ_other_* being an offset.

### 2.5 QSM bench test using QSM2016 challenge 3T data

The performance of the QSM processing was tested on the fully corrected and on the wrapped phase from the QSM2016 challenge data set,^26^ compared with the ground truth susceptibility maps, chi_33. The wrapped phase was unwrapped (Laplacian or ROMEO), and tissue masking was performed using the original mask provided in the challenge and with MM and PB generated from the challenge data. Background removal was performed with V-SHARP (SMV 20 mm) or RESHARP (Tk-12). In addition to iLSQR and MEDI, the closed-form L2-regularized dipole inversion was also used to generate QSM. To obtain *k_Fe_* tissue segmentation and ROI identification was obtained by processing the provided MPRAGE.

## 3 Results

### 3.1 Evaluation of tissue masks

**Figure 1:**
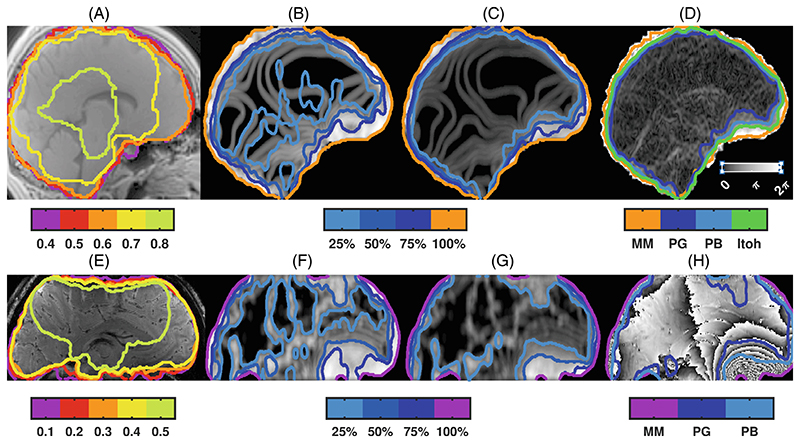
Tissue masks automatically generated from the magnitude alone (MM), or in combination with phase-based masking using the first (PG) or second (PB) derivative of the sign of the wrapped phase. Sagittal views of MRI magnitude images measured with (A) UTE at 3T (TE = 0.1 ms) and (B) with multi-echo GRE at 9.4T after echo summation (TE = 6,12,18,24,30 ms). MM were obtained with BET and different values for the fractional intensity threshold (FIT). To include voxels in the cortex at the brain surface, a FIT = 0.6 was chosen at 3T and 0.1 at 9.4T as comparison basis for the phase-based masks. The sagittal views show PG (B,F) and PB (C,G) obtained from 3T, field map-based simulations (B,C) or experimentally observed at 9.4T,TE = 18 ms (F,G), after smoothing (spherical kernel, radius = 6 voxels). Contour lines indicate thresholding at different levels (25–100%) of the limiting maximum values (*Test^PG^*_max_ and *Test^PB^*_max_, respectively) for each type of smoothing (Supporting Information Figure S3). The norm of the image gradients of the simulated, wrap-free phase image (*Test_Itoh_*) is shown in (D). The ground truth mask containing voxels that fulfill Itoh’s condition (*P_Itoh_*: green contour) is obtained by thresholding *Test_Itoh_* at π. The magnitude (MM, FIT = 0.6, orange contour), and phase-based masks obtained by thresholding at 50% are shown for comparison (PG, light blue contour; PB, dark blue). The corresponding sagittal view, of the wrapped phase data measured in vivo at 9.4T, TE = 18 ms is shown in (H). The MM (FIT = 0.1, magenta contour), PG and PB masks are shown as superimposed contour lines

MM with FIT between 0.4 and 0.6 covered brain tissue voxels at 3T (Figure 1A) while FIT = 0.1 was chosen at 9.4T to include voxels at the brain surface, also in presence of slab selection inhomogeneities (Figure 1E).

Some relevant features of the acquired phase at 9.4T were reproduced by the simulated phase evolution (Supporting Information Figure S2), with dense wraps above the nasal air cavity, especially at late echo times. No large differences between masks were present at 3T or the first two TEs at 9.4T; therefore, the phase at 9.4T, TE = 18 ms was chosen for further analysis.

The shape and size of the smoothing kernel influenced the maximum values of the test function, found in simulations of pure phase noise (Supporting Information Figure S3). For a spherical kernel *Test^PG^_max_* = 6.35 ⋅ *r*^2.87^ (p < 0.001; *R*^2^ = 0.9997) and *Test^PB^_max_* = 6.06 ⋅ *r*^2.84^ (p < 0.001; *R*^2^ = 0.9997). With a Gaussian smoothing kernel, we obtained *Test^PG^_max_* = 1.16 + 0.75 ⋅ *e*^−0.51⋅*FWHM*^ (p < 0.001; *R*^2^ = 0.9998) and *Test^PB^_max_* = 1.04 + 1.30 ⋅ *e*^−0.56⋅*FWHM*^(p < 0.001; *R*^2^ = 0.9986).

Both PG (Figure 1B,F) and PB (Figure 1C,G) showed greater values in brain areas with dense phase wraps. Compared with the ground truth *P_Itoh_* mask (Figure 1D), MM with FIT = 0.6 yielded the highest Dice coefficient (0.91), but also included 6% of voxels outside *P_Itoh_*. For phase-based masking, the Dice coefficient for PB was more similar across thresholds than PG (Supporting Information Figure S3). A spherical kernel (*r* = 6), with 75%(50%) threshold yielded Dice coefficients of 0.92(0.87) for PG and 0.93(0.93) for PB, with 1.4%(0.1%) and 2.0%(0.4%) of voxels violating Itoh’s condition, respectively.

For QSM from the experimental 9.4T data, MM after echo summation with threshold = 0.1, and PB using smoothing with the spherical r = 6 kernel, a threshold value of 500 (51%), followed by image closure and opening using the same kernel were used (Figure 1E-H). The Dice coefficient between MM and PB was 0.96 ± 0.01. Without step 5 to fill holes within the mask and maintain voxels close to the brain surface, the Dice coefficient was 0.97 ± 0.01. A tighter MM was obtained from the single-echo magnitude image and yielded the same Dice coefficient with PB, and 0.97 ± 0.01 with MM.

### 3.2 Effect of masking and background removal on QSM at 9.4T

**Figure 2:**
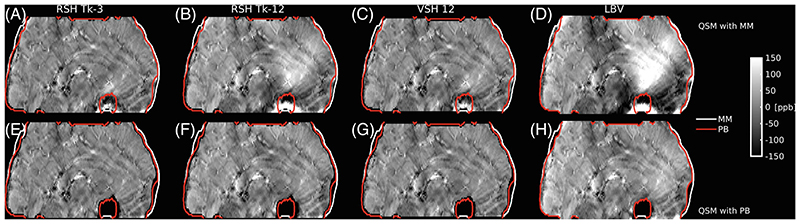
Variation of QSM at 9.4T with different types of tissue masks and background removal, shown as sagittal slices along the midline of a single subject. Results obtained with tissue masks based on magnitude only, MM (A-D), or combined with PB (E-H) are shown for background removal with RESHARP (RSH), a kernel size of 1.6 mm, and a Tikhonov regularization factor of Tk-3 (A,E) or Tk-12 (B,F), or by V-SHARP (VSH) using SMV of 12 mm (C,G) or by LBV (D,H). All background methods except LBV lead to additional mask erosion. QSM using PB generally yielded more homogeneous results across the sagittal slice than MM, except for RSH with Tk-3 and V-SHARP. Results from all 21 subjects using LBV, for which the largest difference between MM and PB was found, are shown in Supporting Information Figure S4

MM worked well in case of RESHARP with high regularization factors (Tk-3, Figure 2A), while less regularization caused strong variations in QSM values especially in the frontal part of the head (Tk-12, Figure 2B). These artifacts were less prominent with V-SHARP SMV (Figure 2C) than with LBV (Figure 2D). Conversely, with PB QSM maps were similar regardless of background removal (Figure 2F-H). The improvement observed varied from subject to subject, depending on brain coverage and head position (Supporting Information Figure S4).

### 3.3 Iron-dependent QSM contrast, *k_Fe_*, at 9.4T

**Figure 3:**
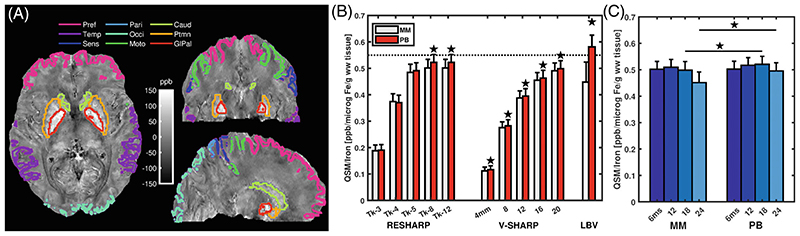
Evaluation of iron-dependent QSM contrast at 9.4T. A, Atlas-based^37^ automatically generated cortical and subcortical brain regions (ROI) are outlined on axial, coronal and sagittal QSM images (scaled between ± 150 ppb) from one subject. B, QSM contrast at TE = 18 ms for different masks (magnitude-only, MM or phase-based, PB) and background removal methods. Results obtained with RESHARP (kernel size: 1.6 mm; Tikhonov regularization between Tk-3 and Tk-12); V-SHARP (SMV between 4-20 mm) and with the Laplacian boundary value (LBV) method with a tolerance of 0.005 are shown. Stronger regularization diminishes QSM contrast, and the greatest contrast is obtained with PB. C, QSM contrast using RESHARP (kernel: 1.6 mm, Tk-12) at different echo times: 6, 12, 18, and 24 ms. The difference between MM and PB was significant (Students’ paired t-test *p* < 0.05) for TE = 18 and 24 ms, indicated with a star. The QSM contrast, *k_Fe_* was obtained as the linear regression coefficient between median QSM values within each ROI, and the age- and ROI-estimated non-heme tissue iron concentration. In (B,C) the average *k_Fe_* across 21 subjects is shown with confidence intervals (*p* < 0.05) indicated by error bars, and the dotted line indicates the lower range value from previous studies.^23,49^ Abbreviations: Pref: prefrontal cortex; Temp: temporal cortex; Sens: primary sensory cortex; Pari: parietal cortex; Occi: occipital cortex; Moto: primary motor cortex; Caud: nucleus caudatus; Ptmn: putamen; GlPal: globus pallidus.

The dependence of QSM values on the age-related estimates of non-heme tissue iron in cortical and subcortical ROIs (Figure 3A) was linear (*p* < 0.001) for all processing pipelines and for each subject, except for LBV using MM for which a significant (*p* < 0.05) linear fit was obtained in 13 out of the 21 subjects. Using MM, the residuals were generally greater in the putamen and caudate than with PB, but the tighter single-echo MM did not lead to a significant change in *k_Fe_* (Student’s paired t-test, T = 1.05, *p* = 0.34).

Significantly greater *k_Fe_* were obtained with PB than with MM, regardless of background removal technique (two-way repeated-measures analysis of variance [ANOVA] F_(1, 2, 20)_=5.99; *p* = 0.024; for RESHARP; F _(1, 2, 20)_ =5.29; *p* = 0.032 for V-SHARP). Pairwise differences were significant for LBV, all VSHARP kernel-sizes, and RESHARP Tk-8, Tk-12 (Figure 3B). Considering all echoes processed with RESHARP Tk-12, there was no significant overall effect, (F_(2,20)_≤1.9; *p* ≥ 0.16), but the pairwise MM-PB differences at TE = 18 ms or more were significant (Figure 3C). No difference between MM and PB was observed with multi-echo combination with an effective TE of 6 ms (Supporting Information Table S2, Supporting Information Figure S7). Combining the latest three echoes (18-30 ms) yielded a lower QSM contrast than 6-18 ms. LBV background removal yielded *k_Fe_* = 0.58 ± 0.10 ppb/μg/g, and 0.60 ± 0.10 when step5 was excluded.

**Figure 4:**
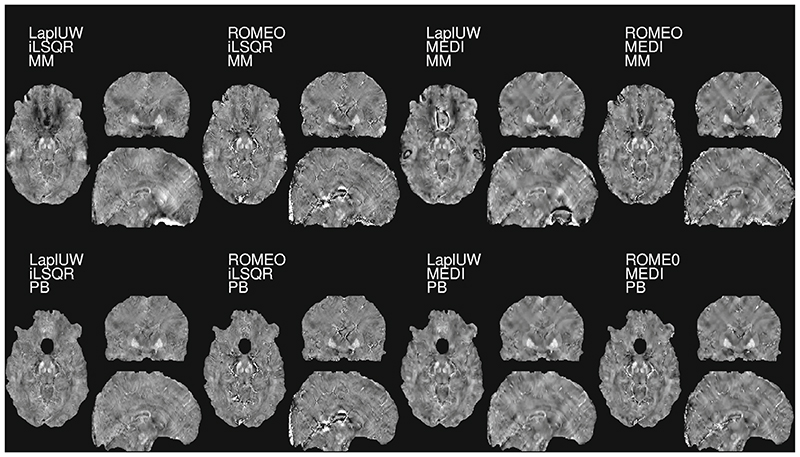
QSM at 9.4T using different masks, unwrapping, and dipole inversion methods but the same background removal (RESHARP, kernel 1.6 mm and Tk-12). Orthonormal views of QSM obtained for one subject with MM and PB masking, different unwrapping (Laplacian or ROMEO) and dipole inversion (iLSQR or MEDI) algorithms are shown. The iron-dependent QSM contrast, *k_Fe_* and offset values *χ_other_* averaged across 21 healthy subjects (age:20–56 y) are tabulated in Supporting Information Table S2. A plot that illustrates the effect of the rapidly varying magnetic field close to the nasal air cavities on the phase wraps, before and after unwrapping and in relation to PB, is shown in Supporting Information Figure S5

Using RESHARP Tk-12 combined with other phase-unwrapping and dipole-inversion algorithms did not influence the average *k_Fe_*, although locally higher QSM values could be observed with ROMEO and MEDI (Figure 4). Close to the nasal air-cavities, the unwrapped phase-values obtained with ROMEO were greater than with the Laplacian approach, while PB successfully identified voxels where discontinuities across the tissue border after unwrapping occurred (Figure S5).

In white matter tracts, PB mitigated the TE dependence of MM, with pronounced effects in the anterior thalamic radiation, the inferior fronto-occipital and the uncinate fasciculi (Supporting Information Figure S8).

### 3.4 Bench test results using QSM2016 challenge 3T data

**Figure 5:**
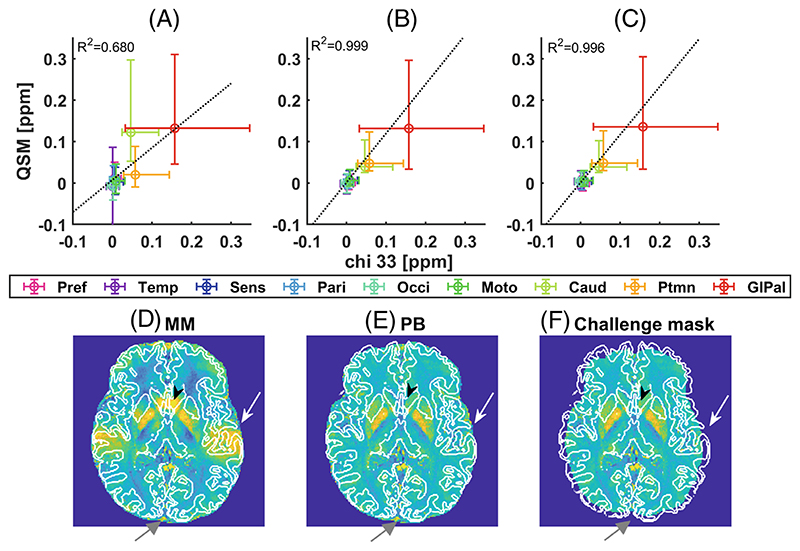
Bench test results obtained from the wrapped phase image provided in the QSM2016 challenge^26^ acquired at 3T after Laplacian unwrapping, RESHARP Tk-12 background removal and iLSQR dipole inversion. QSM values using MM (A), PB (B), or the original mask provided by the QSM2016 challenge (D), obtained within automatically defined subcortical and cortical gray matter regions of interest, are plotted against the ground truth susceptibility values (chi_33). The linear relation between QSM and chi_33 is shown as a dotted black line, and the regression coefficient, R^2^, is shown. Use of MM causes the standard deviation of the QSM values in cortical areas to increase, especially in the temporal cortex above the ear canal (high QSM-values indicated by a white arrow in D-F) and altered QSM-values in the caudate (V-back arrow). These effects are mitigated by PB that yields QSM values which are similar to those obtained with the original challenge mask. Axial QSM images obtained with the three different masks are shown, and the voxels with a gray matter tissue probability > 98% are outlined by a solid white line. The magnitude-based (D) and PB (E) masks both include voxels located at the brain surface which were not included in the original challenge mask (F). The original mask resembles an eroded version of MM (Supporting Information Figure S6). The sagittal vein is maintained in both MM- and PB-processed images but does not cause any QSM artifacts (gray arrow at the bottom). Performance metrics and QSM-contrast obtained using different pipelines on the QSM2016 challenge data are listed in Supporting Information Table S3. Abbreviations: Pref: prefrontal cortex; Temp: temporal cortex; Sens: primary sensory cortex; Pari: parietal cortex; Occi: occipital cortex; Moto: primary motor cortex; Caud: nucleus caudatus; Ptmn: putamen; GlPal: globus pallidus

QSM values obtained with MM, Laplacian unwrapping, RESHARP background removal and iLSQR dipole inversion were slightly lower than the ground truth chi_33 values with MM than with PB or with the original mask provided in the QSM2016 challenge (Figure 5A-C). Both MM and PB included more voxels at the cortical surface than the original mask (Figure 5D-E; Supporting Information Figure S6), but MM yielded increased standard deviations close to air cavities in the caudate and the temporal cortex, which were mitigated by PB. For all processing combinations, MM yielded higher root mean squared error and high frequency error norm values than PB, together with smaller structural similarity index metrics and larger white matter/gray matter errors (Supporting Information Table S3). ROMEO, RESHARP and MEDI dipole inversion using PB yielded a *k_Fe_* which was 0.6% higher than for chi_33.

## 4 Discussion

In the present study, we extended previous QSM work at 3-7T^9,20,21,23,26,50–52^ to 9.4T and developed improved tis sue masks that included voxels at the surface of the brain. Our main finding is that magnitude combined with phase-based masking can be used to reduce artifacts and maintain QSM contrast for different processing pipelines and TEs.

At high magnetic field strengths, when long TEs are used to accommodate high spatial sampling, the effect of background fields at air-tissue interfaces becomes particularly prominent. Densely spaced phase wraps and neighboring voxels with phase differences beyond the limits posed by the ‘Itoh condition’^32^ may occur. In simulations, we observed such effects close to air cavities at long echo times at 9.4T. In experiments, short echo times or combination of multi-echo data to shorten the effective TE, can be envisaged. Nevertheless, depending on the details of head position, phase wraps were present even at TE = 6 ms, calling for the use of inter-echo delays and minimum echo times below this value in future studies.

Our work highlights issues encountered when voxels at the brain boundary are included in the tissue masks, and when QSMs are generated for each TE. This is of interest to study cortical layers in health and disease. In Alzheimer’s disease, QSM shows a cortical layering pattern consistent with β-amyloid accumulation in histology^2^. We showed that MM required the use of strong background filters that diminish the contrast to suppress QSM artifacts. These artifacts could be avoided by exclusion of vulnerable voxels from MM, identified from the phase signal.

The second derivative (PB) of the sign of the wrapped phase yielded robust initial estimates to identify areas with closely spaced wraps and obtain a test function. Smoothing and thresholding at cut-off values that depended on the shape and size of the smoothing kernel was performed to obtain the final mask. These steps could be automatized because of the uniform probability distribution in the presence of pure phase noise, which allowed maximal test function values to be characterized. For instance, a cut-off of 50% can be seen as a threshold for excluding all voxels that are more likely to occur in areas with pure noise or in areas with densely spaced phase wraps where the phase takes on a noise-like behavior.

Regardless of mask, the QSM contrast related to non-heme iron determined at 9.4T was in the lower range of previously observed values between 0.55 and 1.34 ppb/μg/g^24,49^. Field dependence of QSM has been reported previously^53^. Contributing factors for this observation deserve more detailed investigations in future studies. TE-dependent QSM effects have been reported previously and have been ascribed to tissue microstructure.^54^ We found that multi-echo combinations as well as single-echo QSM using PB-masks can diminish TE dependence in gray matter and in white matter fiber tracts. Motion or physiological processes can influence the QSM contrast between the globus pallidum and the frontal cortex by 3–8% at 7T^55^. Such effects deserve to be investigated in future studies.

Phase unwrapping has been recognized as an important cause of underestimation of QSM values, especially for Laplacian based unwrapping^12,15,56^. We found that the use of a quantitative, path-based unwrapping method (ROMEO) overcomes some of these limitations. A restricted axial coverage below 4 cm can lead to underestimations of the QSM contrast^57^ which is however lower than ours (minimum 8 cm). Another factor strongly influencing QSM contrast is background removal. In line with previous studies^18,28^ we found that RESHARP using strong regularization and V-SHARP successfully diminished streaking artifacts that were prominent with MM. However, the QSM contrast was reduced. Using PB, artifacts could be reduced while maintaining a high QSM contrast, regardless of the type of background removal or echo time used. This observation also held true for different dipole inversion algorithms and unwrapping methods. Finally, PB proved useful for evaluating the QSM2016 challenge 3T data when voxels at the brain surface were included.

## 5 Conclusions

Performing QSM with tissue masks which include voxels at the brain boundary derived from the magnitude signal introduces artifacts, hampering quantification. Automatic, phase-based masking founded on the second derivative of the sign of the wrapped phase was able to mitigate artifacts and restore QSM contrast across cortical and subcortical brain regions for different combinations of unwrapping, background removal, and dipole inversion algorithms at different TEs.

## Supplementary Material

Supplementary Material

## Data Availability

Example QSM images obtained with the proposed pipeline at 9.4T and necessary code for generating these images, including extraction of cortical and subcortical ROI data for the QSM2016 challenge data are available on github: ghagberg/PhaseMask4QSM: Phase-based masking for QSM with magnetic resonance imaging (github.com).
