# Supplementary material for: Phase-based masking for quantitative susceptibility mapping of the human brain at 9.4T
Source: Magn Reson Med. Author manuscript; Available in PMC 2023 Nov 1. (PMC7613679; doi:10.1002/mrm.29368)
Supplement: Supplementary Material [file EMS155064-supplement-Supplementary_Material.pdf]

Supporting Information

Phase-based masking for quantitative susceptibility mapping of the human brain at 9.4T

Gisela E Hagberg, Korbinian Eckstein, Elisa Tuzzi, Jiazheng Zhou, Simon Robinson, Klaus Scheffler

Supporting Information Table S1 Parameters A, B and C used to estimate the age-dependent non-haeme tissue iron-content (expressed in [mg iron/100mg wet weight tissue]) [1] in atlas-defined brain regions[2]

**Region HS[1]**	**A**	**B**	**C**	**Regions DK[2]**
Prefrontal cortex	2.43	0.07	0.58	Medial Frontal Gyrus<br>Superior Frontal gyrus
Temporal cortex	2.70	0.07	0.55	Superior Temporal Gyrus, anterior division<br>Superior Temporal Gyrus, posterior division<br>Middle Temporal Gyrus, posterior division<br>Middle Temporal Gyrus, temporo-occipital p.
Sensory cortex	3.97	0.07	0.49	Post-Central Gyrus
Parietal cortex	3.31	0.06	0.60	Superior parietal lobule<br>Supramarginal gyrus, anterior division
Occipital cortex	4.03	0.06	0.72	Lateral occipital cortex<br>Occipital pole
Motor cortex	4.79	0.05	0.40	Pre-central gyrus
Nucleus Caudatus	9.66	0.05	0.33	Caudate
Putamen	14.62	0.04	0.46	Putamen
Globus pallidus	21.41	0.09	0.37	Pallidum

Supporting Information Table S2: Iron-dependent QSM-contrast, k_{Fe}, and offset values, χ_{other} observed in QSM images generated from multi-echo GRE images acquired at 9.4T at single echo times, or using the Fit_ppm_complex_TE[8–11] from the MEDI-toolbox for combinations of 3 or more echoes. If not otherwise stated, unwrapping was performed with the Laplacian method[12], background correction with RESHARP[6](kernel: 1.6mm, Tk -12) and dipole inversion using iLSQR. Note that the result at TE=18ms for this pre-processing pipeline is listed twice (echo-time comparison and comparison of unwrapping/dipole inversion algorithm). The iron concentration was obtained for each subject and region-of-interest (ROI) from the expected age-dependent non-heme iron concentration[1] according to: $[Fe] = A \cdot (1 - e^{-B \cdot age}) + C$, where the ROI-dependent coefficients A, B and C are listed in Supporting Information Table S1. Linear regression analysis: $QSM = k_{Fe}.[Fe] + \chi_{other}$ yielded k_{Fe}, (unit [ppb/µg/g]) to quantify the iron-dependent QSM-contrast, and the offset χ_{other}. Average values and standard deviations across 21 healthy subjects (age:20-56y) are listed.

	**Single-echo**					**Multi-echo**			
	k_{Fe}[ppb/µg/g]		χ_{other}[ppb]			k_{Fe}[ppb/µg/g]		χ_{other}[ppb]	
TE ms	MM	PB	MM	PB	*TE*	MM	PB	MM	PB
6 ms	0.50 (0.07)	0.50 (0.07)	-13.5 (4.0)	-14.1* (4.1)	*6-18 ms*	0.51 (0.08)	0.51 (0.07)	-13.8 (4.0)	-14.4* (3.9)
12 ms	0.51 (0.07)	0.52 (0.07)	-13.9 (3.8)	-15.0* (3.8)	*12-24 ms*	0.48 (0.09)	0.48 (0.08)	-12.9 (4.7)	-13.6 (4.4)
18 ms	0.50 (0.08)	0.52* (0.07)	-13.5 (3.9)	-14.9* (3.9)	*18-30 ms*	0.42 (0.09)	0.43 (0.10)	-10.4 (4.6)	-11.2 (4.2)
24 ms	0.45 (0.09)	0.50* (0.07)	-11.7 (4.1)	-13.8* (3.8)	*6-30 ms*	0.51 (0.08)	0.51 (0.08)	-13.9 (4.3)	-14.4 (4.2)
30 ms	0.37 (0.11)	0.44* (0.08)	- 8.6 (4.7)	-11.4* (4.0)					
	**iLSQR - Laplace**					**MEDI - Laplace**			
	k_{Fe}[ppb/µg/g]		χ_{other}[ppb]			k_{Fe}[ppb/µg/g]		χ_{other}[ppb]	
TE	MM	PB	MM	PB	*TE*	MM	PB	MM	PB
18 ms	0.50 (0.08)	0.52* (0.07)	-13.5 (3.9)	-14.9* (3.9)	*18 ms*	0.54 (0.09)	0.56 (0.09)	-14.8 (6.3)	-15.8* (5.8)
	**iLSQR - ROMEO**					**MEDI - ROMEO**			
	k_{Fe}[ppb/µg/g]		χ_{other}[ppb]			k_{Fe}[ppb/µg/g]		χ_{other}[ppb]	
TE	MM	PB	MM	PB	*TE*	MM	PB	MM	PB
18 ms	0.46 (0.08)	0.46* (0.07)	-13.1 (3.9)	-13.0 (3.7)	*18 ms*	0.51 (0.10)	0.51 (0.10)	-14.6 (6.1)	-15.1 (5.5)

*significant difference between MM and PB, paired T-test, p<0.05

Supporting Information Table S3: QSM performance metrics for QSM2016 challenge[3] 3T data. QSM were generated from the provided tissue phase (Tissue PH), the wrapped phase unwrapped with the Laplacian approach[4] (wPH, Lapl) or with ROMEO[5] (wPH, ROM) after background correction with RESHARP[6] Tk-12 (RSHP) or V-SHARP[7] SMV20mm (VSHP), using different algorithms (closed-form L2-regularized dipole inversion (L2), iLSQR from STI-studio and from the MEDI toolbox) and different masks (magnitude only (MM); phase-based, (PB) and original mask provided by the QSM2016 (Orig)). QSM2016 challenge performance metrics were evaluated: root-mean-squared-error (RSME); high-frequency error norm (HFEN); structural similarity index metric (SSIM); and white-matter-grey-matter error (W+GMerror). In addition, the iron-dependent QSM contrast k_{Fe} across cortical and subcortical brain regions was determined. * k_{Fe} most similar to chi_33; n.s. not-significant linear fit of QSM versus the estimated tissue-iron-concentration

I.nput (Phase/χ)	Dipole InversionAlgorithm	Mask	RSME	HFEN	SSIM	W+GMError	k_{Fe} [ppb/µg/g]
χ, chi_33	n.a. (groundtruth)	Orig	**0.00**	**0.00**	**1.00**	**0.000**	**0.893**
χ, chi_cosmos	n.a.		49	49	0.92	0.013	1.060
Tissue PH	L2		81	75	0.81	0.018	0.866
	iLSQR		97	86	0.78	0.017	0.902
	MEDI		103	94	0.86	0.019	1.037
wPH, Lapl	L2, RSHP	MM	241	130	0.75	0.071	n.s. 0.517
		PB	81	78	0.81	0.021	0.641
		Orig	77	75	0.81	0.021	0.669
	L2, VSHP	MM	150	102	0.76	0.044	n.s. 0.602
		PB	77	76	0.81	0.026	0.593
		Orig	76	74	0.82	0.026	0.610
wPH, Lapl	iLSQR, RSHP	MM	173	124	0.77	0.043	0.779
		PB	99	89	0.79	0.020	0.748
		Orig	97	85	0.80	0.020	0.759
	iLSQR, VSHP	MM	125	107	0.78	0.030	0.807
		PB	96	86	0.79	0.024	0.700
		Orig	94	83	0.80	0.024	0.702
wPH, Lapl	MEDI, RSHP	MM	155	132	0.88	0.022	0.872
		PB	100	94	0.86	0.018	0.851
		Orig	97	91	0.89	0.018	0.882
	MEDI, VSHP	MM	123	110	0.86	0.025	0.937
		PB	94	89	0.85	0.020	0.820
		Orig	92	86	0.85	0.020	0.843
wPH, ROM	L2, RSHP	MM	344	193	0.78	0.110	n.s. 0.293
		PB	88	83	0.80	0.023	0.624
		Orig	78	75	0.81	0.020	0.682
	L2, VSHP	MM	189	129	0.74	0.065	n.s. 0.456
		PB	80	78	0.81	0.026	0.599
		Orig	76	74	0.82	0.026	0.622
wPH, ROM	iLSQR, RSHP	MM	235	191	0.90	0.066	n.s. 0.693
		PB	118	101	0.90	0.022	0.758
		Orig	113	95	0.92	0.020	0.785
	iLSQR, VSHP	MM	169	151	0.92	0.044	0.773
		PB	114	98	0.91	0.024	0.733
		Orig	110	93	0.92	0.024	0.736
wPH, ROM	MEDI, RSHP	MM	195	179	0.93	0.038	0.964
		PB	109	100	0.94	0.019	* 0.888
		Orig	103	94	0.92	0.018	0.917
	MEDI, VSHP	MM	139	128	0.93	0.029	0.998
		PB	103	95	0.94	0.021	0.850
		Orig	98	90	0.91	0.020	0.873

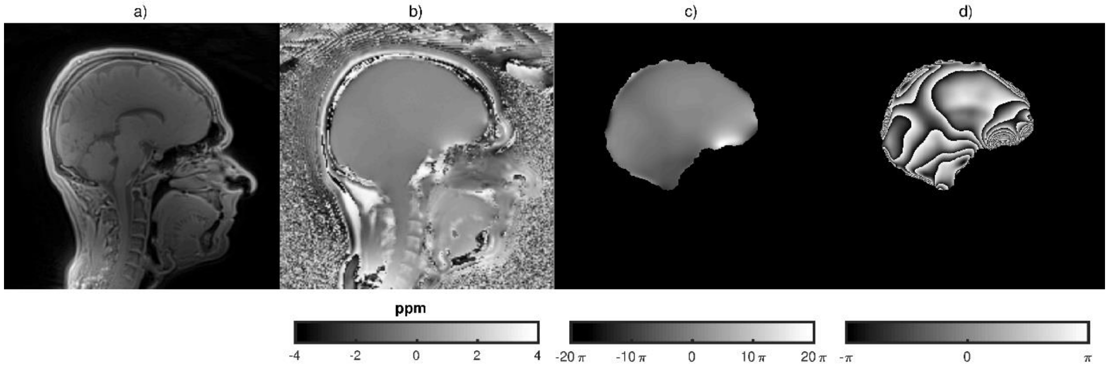

Supporting Information Figure S1: Gradient echo MRI using ultra-short echo (UTE) to obtain field maps at 3T (**a,b**) were used to generate simulated phase images at 9.4T, TE=18ms (**c,d**). Magnitude (**a**) and field map expressed in parts-per-million [ppm] based on the measured field values in Herz and the scanner frequency (**b**). UTE images were obtained at three echo times (TE=0.1, 1.11 and 2.22ms) and voxel sizes of $(1.5\text{mm})^3$. The field map was obtained by spatial upsampling of the phase difference between the first two echoes (**b**). Phase images in radians at 9.4T; TE=18ms; $0.375\text{x}0.375\text{x}0.8\text{mm}^3$ before (**c**) and after (**d**) wrapping into the ±π range are shown.

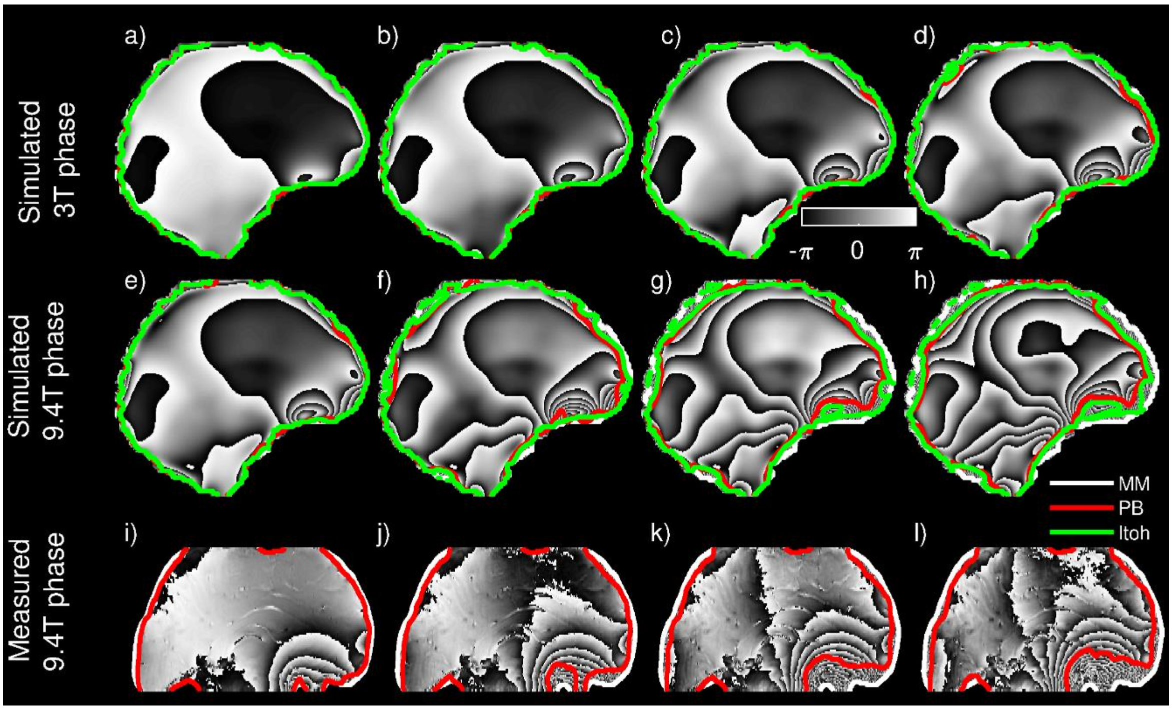

Supporting Information Figure S2: Echo time (TE) dependent evolution of MRI phase images simulated for a mono-polar multi-echo gradient echo sequence at 3T (**a-d**) and 9.4T (**e-h**) and measured in vivo at 9.4T (**i-l**). The wrapped phased in sagittal slices are shown at echo times of 6ms (**a,e,i**) 12ms (**b,f,j**) 18ms (**c,g,k**) and 24ms (**d,h,l**). Automatically generated tissue masks based on the magnitude alone (MM, white solid line) can be compared with phase-based masking (PB, red solid line) and the ground truth mask encompassing voxels where Itoh's condition of neighboring voxels with phase difference <π is fulfilled (green solid lines, simulations only).

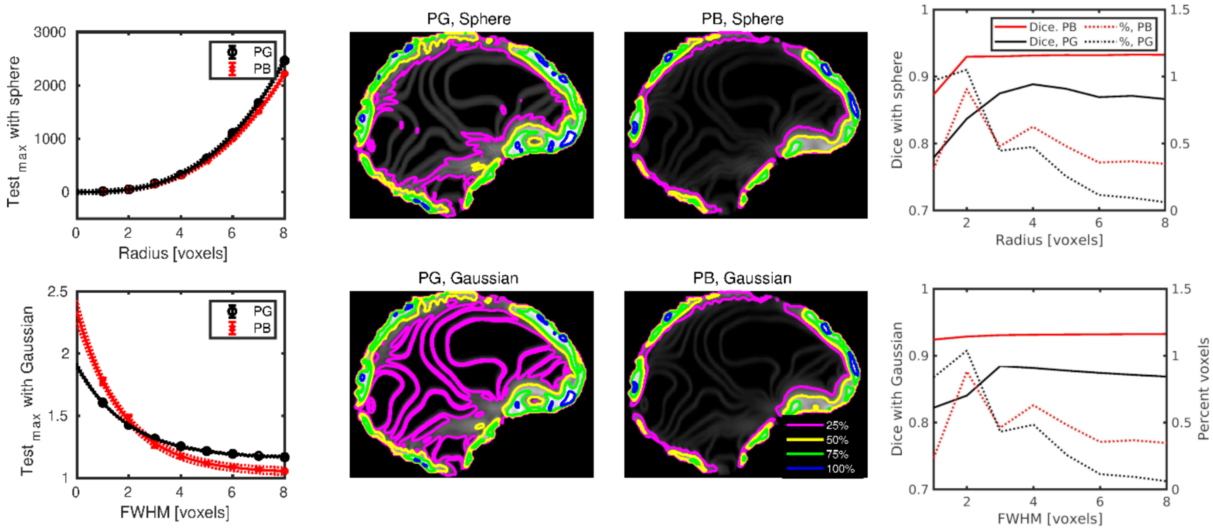

Supporting Information Figure S3: Simulations of the effect of smoothing and thresholding of the test functions to obtain phase-based masks. Test functions were obtained from the sign-function of the wrapped phase, either from its first derivative (PG), or its second derivative (PB), after smoothing (convolution with a spherical kernel, or using a Gaussian function). The expected $Test_{max}$ was obtained from 100 simulations of a 100x100x100 matrix of phase noise for each smoothing kernel and filter width (radius r for the spherical kernel, or full-width-half-maximum $FWHM$ for the Gaussian function). The $Test_{max}$ value was averaged across the 90x90x90 most central voxels each time, and the average and standard deviation across the 100 simulations were plotted against the filter size. For the spherical kernel we found $Test^{PG}{}_{max} = 6.35 \cdot r^{2.87}$ (p<0.001; R^2=0.9997) and $Test^{PB}{}_{max} = 6.06 \cdot r^{2.84}$ (p<0.001; R^2=0.9997). With a Gaussian smoothing kernel, we obtained $Test^{PG}{}_{max} = 1.16 + 0.75 \cdot e^{-0.51 \cdot FWHM}$(p<0.001; R^2=0.9998). $Test^{PB}{}_{max} = 1.04 + 1.30 \cdot e^{-0.56 \cdot FWHM}$(p<0.001; R^2=0.9986).

Simulations from field-maps measured with UTE at 3T data (SupplFig.S1) were used to derive test functions for PG and PB obtained from the wrapped phase at 9.4T with TE=18ms. The sagittal views show results using smoothing with $r = 6$ for the spherical kernel and FWHM=6 for the Gaussian. Thresholding at 25, 50, 75 and 100% of $Test_{max}$ yielded phase-based tissue masks. Masks using different kernel sizes obtained with 50% thresholding were compared with the ground truth mask, containing voxels that fulfill Itoh's condition. Higher Dice coefficients and low values for the percentage of voxels that violated Itoh's conditions were achieved for kernel sizes of 6 and above, while the difference between kernel shapes was negligible. PB-masks generally had higher Dice coefficients, while PG more consistently identified phase wraps, including those in the center of the brain, where unwrapping can be achieved thus leading to erroneous masks at low thresholds.

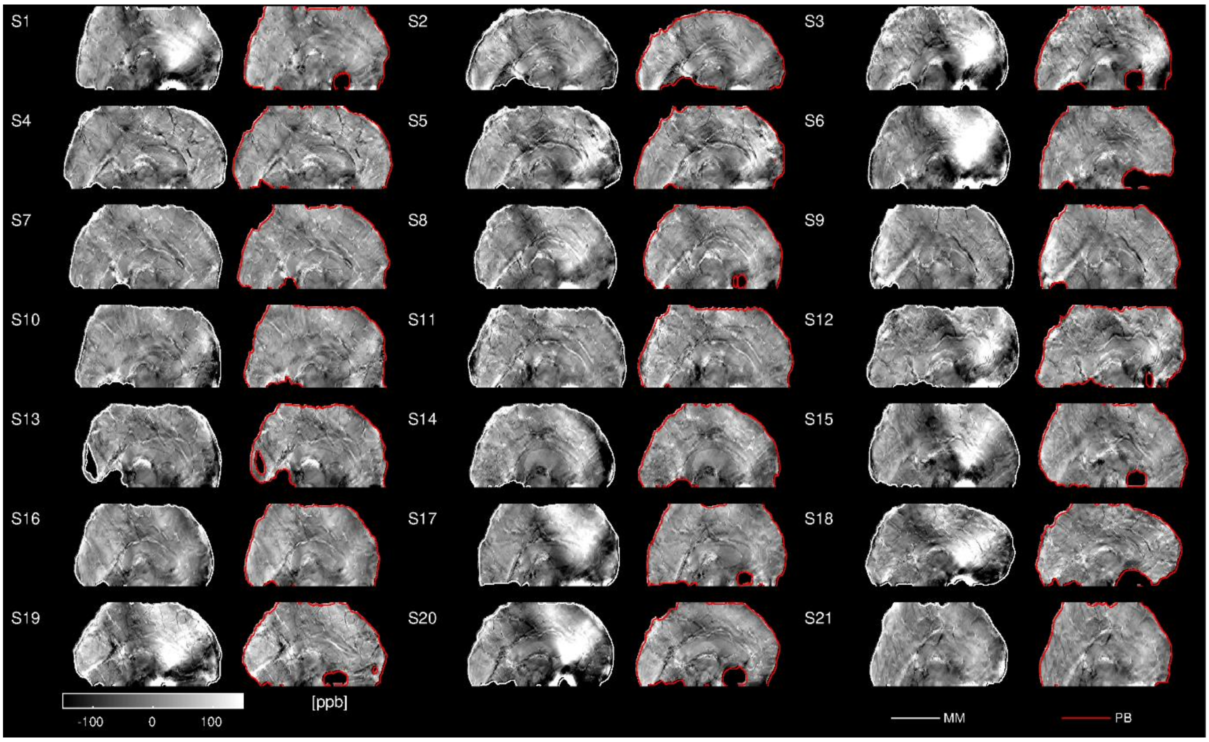

Supporting Information Figure S4: QSM results for 21 subjects measured at 9.4T, TE=18ms, using magnitude (MM, white contour line) and phase (PB, red contour line) masking combined with Laplacian unwrapping[12], LBV-background correction[13] and iLSQR[14]. For each subject and image pair, the same sagittal view along the midline at the center of the brain is shown.

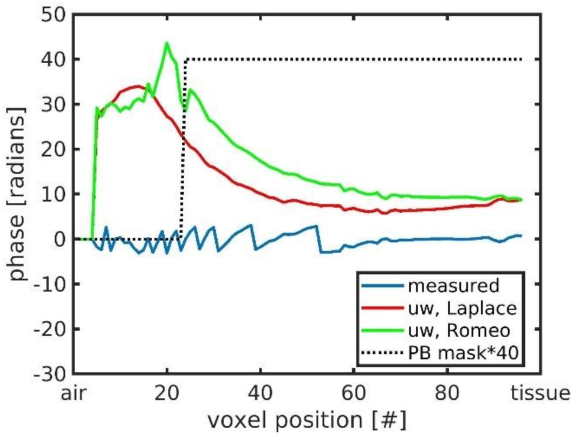

Supporting Information Figure S5. Illustration of the effect of the rapidly varying magnetic field close to the nasal air-cavities on the phase wraps from a healthy volunteer measured at 9.4T wit TE=18ms. The measured phase shows frequent wraps which can be unwrapped using the Laplacian method[8] or Romeo[5]. In the example, the proposed phase based mask, PB, identifies voxels where the wraps were undersampled.

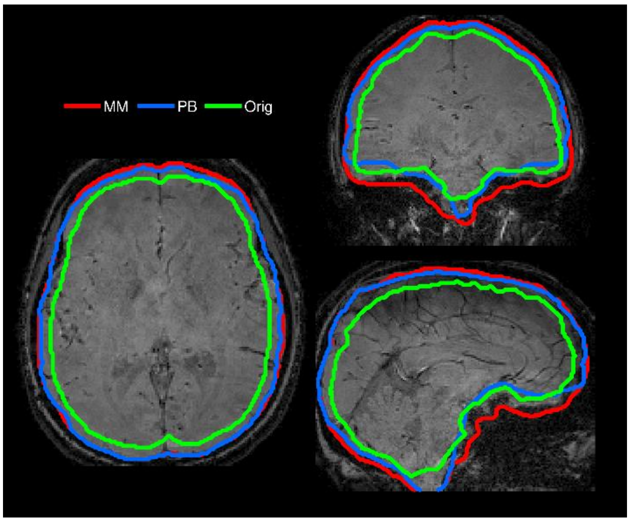

Supporting Information Figure S6. Tissue masks provided (Orig, green solid contour) in the QSM2016 Challenge[3] and generated in the present study using BET[15] on magnitude images only (MM, red contour) and in combination with PB (blue contour).

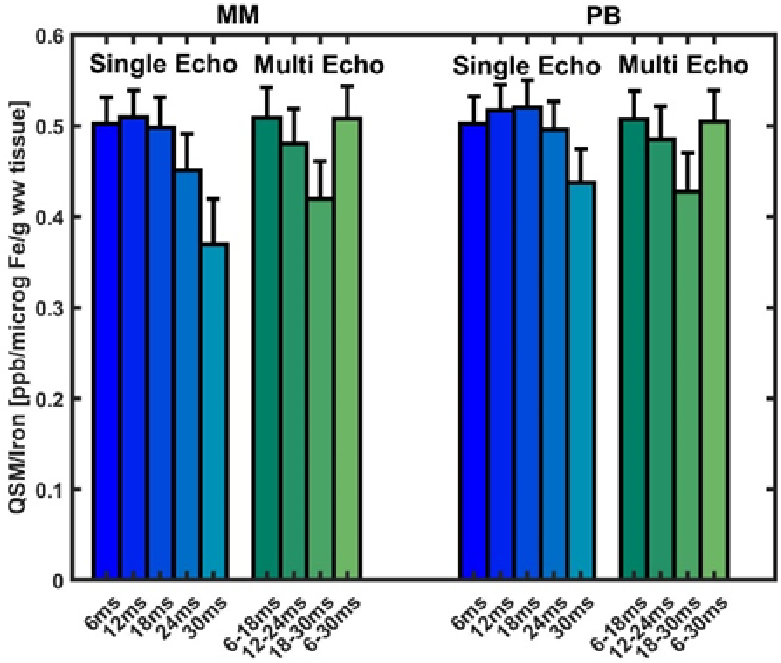

Supporting Information Figure S7. Iron dependent QSM-contrast at 9.4T (average, standard deviation across 21 subjects, cfr Supporting InformationTable S3) obtained with magnitude only (MM) or phase-based (PB) tissue masks in single echo images acquired at TE=6, 12, 18, 24 or 30ms (shades of blue) or for different combinations of multi-echo images with an inter-echo delay of 6ms (shades of green). Either tree echoes between 6-18ms; 12-24ms; or 18-30ms or all five echoes between 6-30ms were used for the echo combination based on the Fit_ppm_TE_complex available in MEDI, as described previously[16].

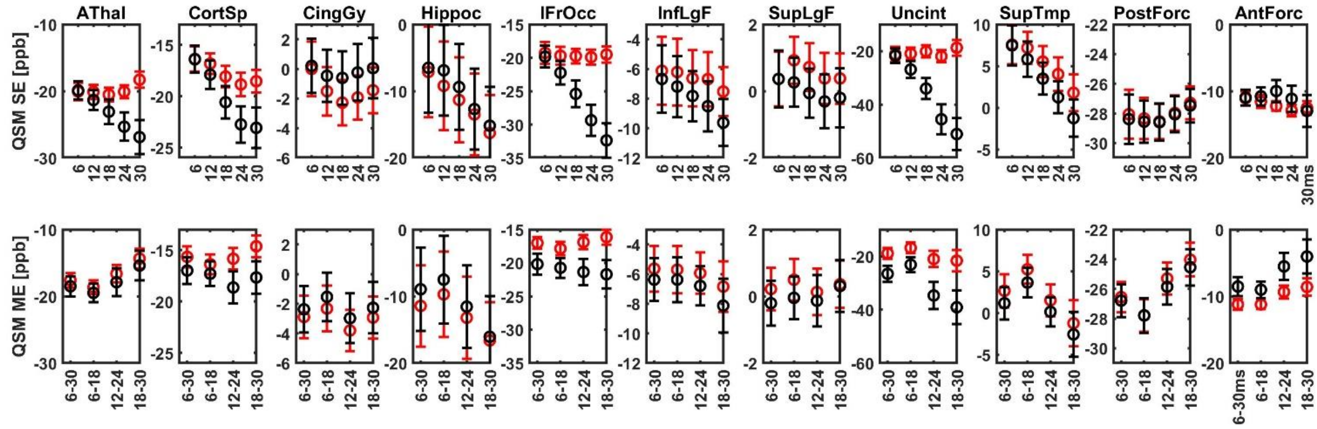

Supporting Information Figure S8. QSM values at 9.4T in white matter fibre tracts obtained from the JHU-atlas[17] (probability threshold: 25%). Values were obtained from single echo based QSM (SE, top) or from QSM obtained using different combinations of multi echo data (ME, bottom, cfr Fig. S7) using magnitude only (MM) or phase (PB) masks (average, standard deviation across 21 subjects). Except for the forceps, results in the left and right hemisphere were averaged. Abbreviations: AThal: anterior thalamic radiation; CortSp: corticospinal tract; CingGy: cingulate gyrus; Hippoc: hippocampus; IFrOcc: inferior fronto-occipital fasciculus; InfLgF: inferior longitudinal fasciculus; SupLg: superior longitudinal fasciculus; Uncint: uncinate fasciculis; SupTmp: temporal part of SupLg; PostForc: posterior forceps (forceps major) with tracts through the splenium of the corpus callosum; AntForc: anterior forceps (forceps minor) with tracts through the genu of the corpus callosum.

References

1. Hallgren B, Sourander P. The effect of age on the non-haemin iron in the human brain. *J Neurochem*. 1958;3(1):41-51. doi:10.1111/j.1471-4159.1958.tb12607.x

2. Desikan RS, Ségonne F, Fischl B, et al. An automated labeling system for subdividing the human cerebral cortex on MRI scans into gyral based regions of interest. *Neuroimage*. 2006;31(3):968-980. doi:10.1016/j.neuroimage.2006.01.021

3. Langkammer C, Schweser F, Shmueli K, et al. Quantitative susceptibility mapping: Report from the 2016 reconstruction challenge. *Magn Reson Med*. 2018;79(3):1661-1673. doi:10.1002/mrm.26830

4. Liu Z, Kee Y, Zhou D, Wang Y, Spincemaille P. Preconditioned total field inversion (TFI) method for quantitative susceptibility mapping. *Magn Reson Med*. 2017;78(1):303-315. doi:10.1002/mrm.26331

5. Dymerska B, Eckstein K, Bachrata B, et al. Phase unwrapping with a rapid opensource minimum spanning tree algorithm (ROMEO). *Magn Reson Med*. 2021;85(4):2294-2308. doi:10.1002/mrm.28563

6. Sun H, Wilman AH. Background field removal using spherical mean value filtering and Tikhonov regularization. *Magn Reson Med*. 2014;71(3):1151-1157. doi:10.1002/mrm.24765

7. Li W, Wu B, Liu C. Quantitative susceptibility mapping of human brain reflects spatial variation in tissue composition. *Neuroimage*. 2011;55(4):1645-1656. doi:10.1016/j.neuroimage.2010.11.088

8. Liu T, Wisnieff C, Lou M, Chen W, Spincemaille P, Wang Y. Nonlinear formulation of the magnetic field to source relationship for robust quantitative susceptibility mapping. *Magn Reson Med*. 2013;69(2):467-476. doi:10.1002/mrm.24272

9. Kressler B, de Rochefort L, Liu T, Spincemaille P, Jiang Q, Wang Y. Nonlinear regularization for per voxel estimation of magnetic susceptibility distributions from MRI field maps. *IEEE Trans Med*

Imaging. 2010;29(2):273-281. doi:10.1109/TMI.2009.2023787

10. de Rochefort L, Brown R, Prince MR, Wang Y. Quantitative MR susceptibility mapping using piece-wise constant regularized inversion of the magnetic field. *Magn Reson Med*. 2008;60(4):1003-1009. doi:10.1002/mrm.21710

11. Bernstein MA, Grgic M, Brosnan TJ, Pelc NJ. Reconstructions of phase contrast, phased array multicoil data. *Magn Reson Med*. 1994;32(3):330-334. doi:10.1002/mrm.1910320308

12. Schofield MA, Zhu Y. Fast phase unwrapping algorithm for interferometric applications. *Opt Lett*. 2003;28(14):1194-1196. doi:10.1364/ol.28.001194

13. Zhou D, Liu T, Spincemaille P, Wang Y. Background field removal by solving the Laplacian boundary value problem. *NMR Biomed*. 2014;27(3):312-319. doi:10.1002/nbm.3064

14. Li W, Wang N, Yu F, et al. A method for estimating and removing streaking artifacts in quantitative susceptibility mapping. *Neuroimage*. 2015;108:111-122. doi:10.1016/j.neuroimage.2014.12.043

15. Smith SM. Fast robust automated brain extraction. *Hum Brain Mapp*. 2002;17(3):143-155. doi:10.1002/hbm.10062

16. Hagberg GE, Eckstein K, Cuna E, Robinson S, Scheffler K. Towards robust QSM in cortical and sub-cortical regions of the human brain at 9.4T: influence of coil combination and masking strategies. *Proc Intl Soc Mag Reson Med*. 2020;28:3786. https://index.mirasmart.com/ISMRM2020/PDFfiles/3786.html

17. Hua K, Zhang J, Wakana S, et al. Tract probability maps in stereotaxic spaces: Analyses of white matter anatomy and tract-specific quantification. *Neuroimage*. 2008;39(1):336-347. doi:10.1016/j.neuroimage.2007.07.053
